# Risk factors for postoperative adverse outcomes in patients with high anal fistula undergoing modified TROPIS procedure combined with Parks’ fistulotomy with seton: a retrospective study

**DOI:** 10.3389/fmed.2026.1834676

**Published:** 2026-06-01

**Authors:** Benfan Yin, Jiaying Li, Buqiang Chen, Xingwang Liang

**Affiliations:** 1 Department of Anorectal Medicine, Seventh People’s Hospital of Shanghai University of Traditional Chinese Medicine, Shanghai, China; 2 Department of Traditional Chinese Medicine, Seventh People’s Hospital of Shanghai University of Traditional Chinese Medicine, Shanghai, China

**Keywords:** high anal fistula, Parks’ fistulotomy, postoperative outcomes, prediction model, risk factors, seton, TROPIS procedure

## Abstract

**Background:**

High anal fistula poses therapeutic challenges, with traditional fistulotomy associated with 20–50% recurrence and incontinence risk. This study evaluated adverse outcomes and risk factors in patients undergoing modified TROPIS combined with Parks’ fistulotomy and seton.

**Methods:**

This retrospective cohort study included 218 consecutive patients who underwent modified TROPIS with Parks’ fistulotomy and seton for high anal fistula between May 2022 and May 2024. The primary outcome was postoperative adverse outcome (PAO), a composite endpoint encompassing recurrence, anal incontinence, delayed wound healing, and major complications within 6 months. Risk factors were identified using Firth’s penalized logistic regression with bootstrap internal validation.

**Results:**

Postoperative adverse outcomes occurred in 49 patients (22.5%): recurrence in 8.3%, incontinence in 6.9%, delayed healing in 10.1%, and major complications in 3.7%. Five independent risk factors were identified: diabetes mellitus (adjusted OR 3.42, 95% CI 1.58–7.41), horseshoe extension (OR 2.98, 95% CI 1.45–6.12), internal sphincter incision >50% circumference (OR 2.76, 95% CI 1.32–5.78), operative time >60 min (OR 2.35, 95% CI 1.12–4.93), and BMI ≥ 28 kg/m^2^ (OR 2.08, 95% CI 1.05–4.12). Model B demonstrated good discrimination (C-statistic 0.82, 95% CI 0.76–0.88; optimism-corrected 0.80) and outperformed Model A (0.74; *p* = 0.008). Risk stratification identified 31.2% as high-risk (predicted probability >30%), with observed adverse outcome rates of 41.2% versus 14.0% in low-risk patients (*p* < 0.001).

**Conclusion:**

Modified TROPIS with Parks’ fistulotomy and seton for high anal fistula carries a 22.5% adverse outcome rate, predicted by diabetes, horseshoe extension, sphincter division >50%, operative time >60 min, and obesity. These findings require external validation in a larger cohort before clinical implementation.

## 1 Introduction

High anal fistula, defined as those traversing the anorectal ring or involving the supralevator space, poses a fundamental therapeutic dilemma: radical eradication must be balanced against preservation of anal continence (1, 2). Traditional fistulotomy, while effective for simple fistulas, is associated with recurrence rates of 20–50% and significant incontinence risk in high fistulas, profoundly impacting patient quality of life (3). This limitation has driven the development of sphincter-preserving techniques, including the transanal opening of intersphincteric space (TROPIS) procedure and Parks’ fistulotomy with seton drainage (4, 5).

Modified TROPIS combined with Parks’ fistulotomy and seton represents an evolving strategy that addresses the internal opening through controlled intersphincteric dissection while managing the transsphincteric component via gradual drainage (6, 7). Although preliminary institutional experiences suggest feasibility, the safety profile and risk factors for adverse outcomes following this combined approach remain poorly characterized. Existing literature has identified diabetes, horseshoe extension, and previous surgery as predictors of failure following individual procedures (8, 9); however, these findings may not be generalizable to combined techniques with distinct technical considerations. Moreover, most previous investigations have examined recurrence or incontinence in isolation, potentially overlooking the interconnected nature of adverse outcomes in complex fistula surgery (10).

Therefore, this study aimed to: (i) determine the incidence of composite postoperative adverse outcomes—encompassing recurrence, anal incontinence, delayed wound healing, and major complications within 6 months—in patients undergoing modified TROPIS combined with Parks’ fistulotomy with seton for high anal fistula; and (ii) identify independent preoperative and intraoperative risk factors and develop a clinically applicable prediction model for risk stratification.

## 2 Materials and methods

### 2.1 Study design and ethical approval

This retrospective cohort study followed the Strengthening the Reporting of Observational Studies in Epidemiology (STROBE) guidelines. The Institutional Review Board (IRB) of Seventh People’s Hospital of Shanghai University of Traditional Chinese Medicine approved the protocol (Approval No: KY2024025016). Given the retrospective design, the IRB waived informed consent; all data were anonymized before analysis.

### 2.2 Study population and setting

Consecutive patients who underwent modified TROPIS combined with Parks’ fistulotomy with seton for high anal fistula at our hospital between May 2022 and May 2024 were reviewed.

(i) age ≥18 years; (ii) high anal fistula confirmed by preoperative MRI or EAUS, defined as fistula crossing the anorectal ring (proximal boundary of the puborectalis muscle and external sphincter complex); (iii) Parks’ transsphincteric/suprasphincteric or Garg grade IIIA–IV; (iv) internal opening located ≥2 cm from the anal verge with tract traversing ≥30% of the external sphincter thickness on axial T2-weighted MRI; (v) complete perioperative records and ≥6-month follow-up; (vi) preoperative and 6-month functional assessments available.

*Exclusion criteria*: (i) intersphincteric or superficial fistulas; (ii) Crohn’s disease or inflammatory bowel disease; (iii) previous anal surgery for the same fistula; (iv) rectovaginal/rectourethral fistula; (v) active perianal abscess requiring emergency drainage; (vi) anorectal malignancy; (vii) pregnancy or lactation; (viii) immunosuppressive therapy or severe immunodeficiency; (ix) incomplete records or loss to follow-up (<6 months).

### 2.3 Surgical procedure

Procedures were performed by two surgeons (X. L.: *n* = 112, B. C.: *n* = 106) with comparable experience (>10 years, >500 TROPIS procedures each). Complex cases were equally distributed between surgeons. The choice of incision extent and seton type was determined by the operating surgeon based on intraoperative findings, with complex cases discussed jointly preoperatively. All patients underwent the modified TROPIS procedure combined with Parks’ fistulotomy with seton under general anesthesia in the lithotomy position. The operative technique involved: (1) identification of the internal opening via anoscopy and probe insertion; (2) transanal opening of the intersphincteric space with partial division of the internal sphincter muscle while preserving the external sphincter; (3) fistulotomy of the superficial portion of the fistula tract; (4) placement of a loose seton (silastic vessel loop or silk suture) through the remaining deep tract for dependent drainage; and (5) curettage of granulation tissue within the fistula tract. The extent of internal sphincter division and seton tension were determined by the operating surgeon based on fistula complexity and intraoperative findings.

The modified TROPIS procedure differed from the original Garg technique in that: (i) the intersphincteric incision was limited to ≤40% of the internal sphincter circumference in principle; (ii) the internal opening was primarily closed with absorbable sutures (Vicryl 3–0) when tension-free, otherwise left open; and (iii) concomitant seton placement through the transsphincteric component was performed in all cases. These modifications aim to reduce sphincter division-related incontinence while maintaining drainage efficacy, differing from the original Garg technique which relied solely on intersphincteric dissection without predefined sphincter preservation limits. However, in cases with extensive horseshoe extension, supralevator involvement, or dense fibrosis preventing adequate drainage, the incision was extended beyond 40% to achieve complete fistula eradication, with the extent of division recorded prospectively. Tightening seton was used when >30% external sphincter was traversed or manometry showed resting pressure <40 mmHg; loose seton for <30% involvement with normal pressure. Seton indication was recorded to assess confounding.

### 2.4 Data collection and variables

Comprehensive clinical data were extracted from electronic medical records, operative reports, imaging databases, and prospective functional assessment registries by two independent researchers using a standardized data collection form. Discrepancies were resolved by consensus or consultation with a third investigator.

Demographic and clinical variables included age, sex, body mass index (BMI), smoking status, comorbidities (diabetes mellitus, hypertension, cardiovascular disease), and preoperative glycemic control (HbA1c for diabetic patients).

Fistula-related characteristics comprised Parks’ classification (transsphincteric vs. suprasphincteric), Garg grading (IIIA/IIIB/IV), fistula duration (months), previous perianal abscess history, horseshoe extension (circular involvement of anal canal), number of fistula tracts (single vs. multiple), internal opening location (posterior vs. lateral/anterior), internal opening height (distance from anal verge in cm), presence of secondary extensions (supralevator or ischiorectal fossa involvement), and preoperative seton drainage. Preoperative fistula complexity was objectively assessed using the modified Van Assche MRI index.

Perioperative indicators included operative time (minutes), estimated blood loss (mL), extent of internal sphincter incision (cm and percentage of circumference), seton type (tightening vs. loose), seton material, number of setons placed, perioperative antibiotic protocols (preoperative antibiotic duration for active infection, postoperative intravenous and oral antibiotic regimen and duration), postoperative antibiotic duration (days), hospital stay (days), and time to seton removal (days). Perioperative antibiotic protocols were standardized across all patients. Intravenous prophylaxis was administered within 60 min of incision, typically ceftriaxone 2 g plus metronidazole 500 mg (or ciprofloxacin 400 mg plus metronidazole 500 mg for penicillin-allergic patients). For patients with active perianal infection at presentation, preoperative antibiotics were continued until clinical resolution. Postoperatively, intravenous antibiotics were continued for 24–48 h, followed by oral antibiotics (metronidazole 400 mg three times daily plus ciprofloxacin 500 mg twice daily, or amoxicillin-clavulanate 625 mg three times daily) until seton removal or wound epithelialization. Total postoperative antibiotic duration was recorded.

### 2.5 Outcome definitions and assessment

The primary outcome was postoperative adverse outcome (PAO), defined as a composite endpoint occurring within 6 months postoperatively, including any of the following: (i) recurrence: reappearance of fistula symptoms with confirmation by MRI or EAUS interpreted by radiologists blinded to surgical details, or need for reoperation; (ii) anal incontinence: moderate to severe fecal incontinence defined as Wexner incontinence score ≥7 points, assessed by assessor-blinded independent research coordinators who were not involved in clinical care and had no access to operative records; patients inherently knew their symptoms, but interviewers followed structured scripts; (iii) delayed wound healing: failure of complete wound epithelialization at 3-month postoperative examination assessed by surgeons, with photographs reviewed by an independent observer blinded to outcomes; and (iv) major complications: Grade III or higher complications according to the Clavien-Dindo classification (objective events requiring intervention).

Secondary outcomes included: (i) changes in Wexner incontinence score from preoperatively to 6 months postoperatively; (ii) anorectal manometry parameters (anal resting pressure, maximal squeeze pressure, sphincter functional length) measured preoperatively and at 6 months by a single experienced technician blinded to surgical details; (iii) quality of life assessed by the Quality of Life Assessment for Fistula Questionnaire Score (QLAF-QS) administered by research staff independent of the surgical team; and (iv) wound healing time, postoperative pain visual analog scale (VAS) scores, and patient satisfaction scores collected via standardized questionnaires.

### 2.6 Follow-up protocol

Patients were followed up at 1 week, 1 month, 3 months, and 6 months postoperatively. Clinical examination was performed at each visit to assess wound healing, seton status, and continence. The Wexner incontinence score was recorded preoperatively and at 6 months. Anorectal manometry was performed preoperatively and repeated at 6 months by a single experienced technician blinded to the surgical details. MRI was repeated at 6 months or earlier if recurrence was suspected based on clinical findings.

All patients followed a standardized care pathway: (1) Wound irrigation with povidone-iodine solution (1:10 dilution) twice daily beginning postoperative day 1; (2) Sitz baths (warm water, 10–15 min) three times daily; (3) Stool softeners (polyethylene glycol 4,000, 10 g twice daily) for 4 weeks; (4) Fiber supplementation (psyllium husk, 3.5 g twice daily) to achieve soft-formed stools; (5) Weekly outpatient seton adjustment until removal; (6) Prohibited activities: heavy lifting >10 kg and cycling for 6 weeks. Analgesia followed a stepwise protocol (paracetamol 1 g q6h → tramadol 50 mg q8h if VAS > 4). Compliance was assessed via patient diaries and reviewed at each follow-up visit.

### 2.7 Statistical analysis

Given the retrospective design, we included all eligible consecutive patients (*n* = 218) during the study period. With 49 adverse outcome events and 7 pre-specified variables in the final multivariable model, the EPV ratio was 7.0, below the conventional threshold of 10 events per variable required for stable logistic regression estimates. To mitigate finite-sample bias, we employed Firth’s penalized likelihood logistic regression with Jeffreys prior penalization and performed bootstrap internal validation (B = 1,000) to quantify model optimism. Effect estimates are presented with 95% confidence intervals to reflect uncertainty. All variables in the primary multivariable model (demographics, fistula characteristics, perioperative indicators, and primary outcome) had complete data for all 218 patients. Missing data were limited to secondary functional outcomes: 6-month anorectal manometry (*n* = 7, 3.2%), Wexner incontinence score (*n* = 5, 2.3%), and quality-of-life questionnaires (*n* = 6, 2.8%), due to missed follow-up visits. Given the low missingness rate (<5% for any variable) and the absence of missing data in primary model variables, complete case analysis was used as the primary approach for all analyses. Multiple imputation was not performed.

Statistical analyses were performed using R version 4.3.0. Continuous variables were assessed for normality using the Shapiro–Wilk test and expressed as mean ± standard deviation or median (interquartile range) as appropriate; categorical variables were expressed as frequencies (%). Baseline characteristics were compared between patients with and without adverse outcomes using independent *t*-test or Mann–Whitney U test for continuous variables, and Chi-square or Fisher’s exact test for categorical variables. Variables with *p* < 0.10 in univariate analysis were entered into multivariate logistic regression with backward stepwise selection to identify independent risk factors. Seton type was forced into the final model given its clinical relevance, despite potential confounding by indication. The final model retained variables with *p* < 0.05, with results expressed as adjusted odds ratios (OR) and 95% confidence intervals (CI). Model discrimination was evaluated using the C-statistic, calibration by the Hosmer-Lemeshow test, and collinearity by variance inflation factor (VIF); variables with VIF > 5 were excluded or combined. Clinically relevant interaction terms were tested. Two prediction models were developed: Model A (preoperative variables only) and Model B (preoperative plus intraoperative variables). Bootstrap internal validation (B = 1,000) was performed to estimate optimism-corrected performance. For functional outcomes, pre- and postoperative Wexner scores and manometry parameters were compared using paired *t*-test or Wilcoxon signed-rank test. Correlations between anatomical variables and functional outcomes were assessed using Pearson or Spearman coefficients. Sensitivity analyses included: (i) excluding diabetic patients; (ii) alternative incontinence definitions (Wexner score ≥5 vs. ≥7); (iii) composite endpoint excluding incontinence; and (iv) competing-risk analysis for individual outcomes. A two-sided *p* < 0.05 was considered statistically significant.

## 3 Results

### 3.1 Patient characteristics and follow-up

**Figure 1:**
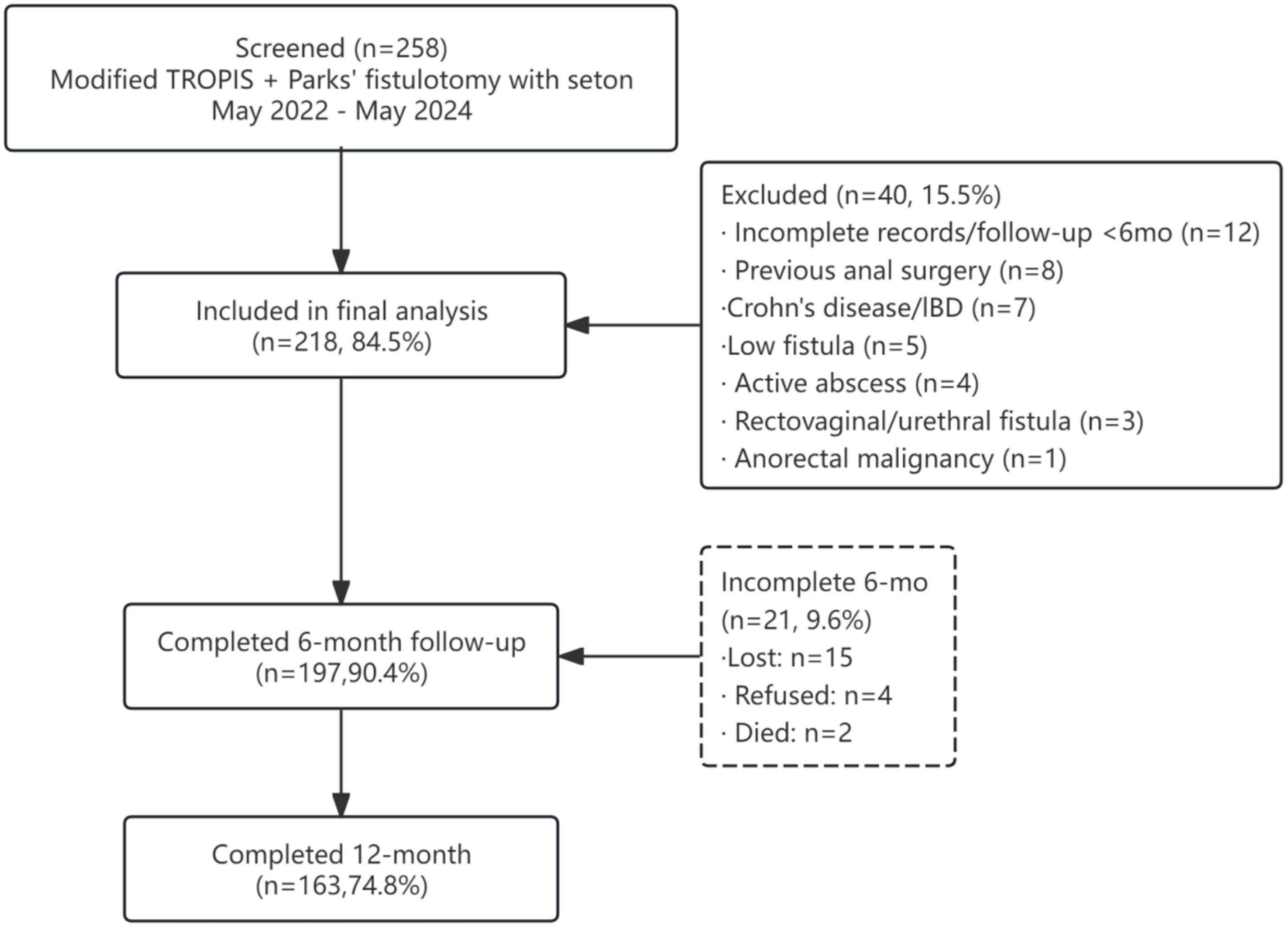
Flow diagram of patient selection.

Between May 2022 and May 2024, 258 consecutive patients underwent modified TROPIS with Parks’ fistulotomy and seton placement for high anal fistula. After excluding 40 patients (15.5%) for incomplete follow-up (*n* = 12), prior anal surgery (*n* = 8), inflammatory bowel disease (*n* = 7), low fistula anatomy (*n* = 5), active abscess (*n* = 4), rectovaginal/urethral fistula (*n* = 3), or anorectal malignancy (*n* = 1), 218 patients (84.5%) were included in the final analysis (Figure 1).

Postoperative adverse outcomes occurred in 49 patients (22.5%). Patients in the PAO-positive group were significantly older (48.2 ± 13.1 vs. 40.9 ± 11.8 years, *p* < 0.001) and had greater metabolic burden, evidenced by higher BMI (26.4 ± 3.8 vs. 24.3 ± 3.1 kg/m^2^, *p* = 0.002), higher obesity rates (36.7% vs. 17.8%, *p* = 0.006), and more prevalent diabetes mellitus (34.7% vs. 12.4%, *p* < 0.001), particularly with poor glycemic control (HbA1c ≥ 8.0%: 18.4% vs. 4.1%, *p* < 0.001). Smoking history was also more common in this group (36.7% vs. 22.5%, *p* = 0.042).

Regarding fistula characteristics, PAO-positive patients presented with more complex anatomy and longer disease duration (median 12.3 vs. 7.2 months, *p* = 0.008). Specifically, they exhibited higher rates of horseshoe extension (42.9% vs. 15.4%, *p* < 0.001), multiple fistula tracts (51.0% vs. 27.2%, *p* = 0.003), supralevator involvement (24.5% vs. 9.5%, *p* = 0.003), internal openings located >3 cm from the anal verge (38.8% vs. 19.5%, *p* = 0.003), and advanced Garg grading (grade IV: 28.6% vs. 10.1%, *p* = 0.002). The modified Van Assche MRI index was correspondingly elevated in these patients (median 10.5 vs. 7.2, *p* = 0.002). Notably, Parks’ classification and hypertension showed no significant between-group differences (Table 1).

**Table 1:** Baseline demographic and fistula-related characteristics.

Variable	Total (*n* = 218)	PAO-positive (*n* = 49)	PAO-negative (*n* = 169)	*p*-value
Demographics				
Age, years, mean ± SD	42.6 ± 12.3	48.2 ± 13.1	40.9 ± 11.8	<0.001
Sex, male, *n* (%)	172 (78.9)	41 (83.7)	131 (77.5)	0.32
BMI, kg/m^2^, mean ± SD	24.8 ± 3.4	26.4 ± 3.8	24.3 ± 3.1	0.002
BMI ≥ 28 kg/m^2^, *n* (%)	48 (22.0)	18 (36.7)	30 (17.8)	0.006
Current/former smoker, *n* (%)	56 (25.7)	18 (36.7)	38 (22.5)	0.042
Comorbidities				
Diabetes mellitus, *n* (%)	38 (17.4)	17 (34.7)	21 (12.4)	<0.001
HbA1c ≥ 8.0%, *n* (%)	16 (7.3)	9 (18.4)	7 (4.1)	<0.001
HbA1c < 8.0%, *n* (%)	22 (10.1)	8 (16.3)	14 (8.3)	0.048
Hypertension, *n* (%)	42 (19.3)	14 (28.6)	28 (16.6)	0.045
Cardiovascular disease, *n* (%)	18 (8.3)	7 (14.3)	11 (6.5)	0.058
Fistula characteristics				
Fistula duration, months, median (IQR)	8.5 (4.2–15.6)	12.3 (6.8–20.4)	7.2 (3.8–13.2)	0.008
Previous perianal abscess, *n* (%)	89 (40.8)	26 (53.1)	63 (37.3)	0.048
Parks’ classification, *n* (%)				0.28
Transsphincteric	156 (71.6)	32 (65.3)	124 (73.4)	
Suprasphincteric	62 (28.4)	17 (34.7)	45 (26.6)	
Garg grading, *n* (%)				
Grade IIIA	89 (40.8)	15 (30.6)	74 (43.8)	0.11
Grade IIIB	98 (45.0)	20 (40.8)	78 (46.2)	0.52
Grade IV	31 (14.2)	14 (28.6)	17 (10.1)	0.002
Horseshoe extension, *n* (%)	47 (21.6)	21 (42.9)	26 (15.4)	<0.001
Multiple fistula tracts, *n* (%)	71 (32.6)	25 (51.0)	46 (27.2)	0.003
Internal opening >3 cm from verge, *n* (%)	52 (23.9)	19 (38.8)	33 (19.5)	0.003
Supralevator extension, *n* (%)	28 (12.8)	12 (24.5)	16 (9.5)	0.003
Preoperative seton drainage, *n* (%)	45 (20.6)	14 (28.6)	31 (18.3)	0.12
Modified Van Assche index, median (IQR)	8.0 (5.2–11.5)	10.5 (7.2–14.8)	7.2 (4.8–10.2)	0.002

PAO, postoperative adverse outcome; BMI, body mass index; IQR, interquartile range. Continuous variables compared using independent *t*-test or Mann–Whitney U test. Categorical variables compared using Chi-square test or Fisher’s exact test. All patients met criteria for high fistula (crossing anorectal ring) by MRI/EAUS. Parks classification and Garg grade were both recorded for descriptive completeness; Garg grade was selected for multivariable analysis due to stronger univariate association and finer stratification of complexity.

### 3.2 Perioperative characteristics

All patients received perioperative antibiotics per standardized protocol. Preoperative antibiotics for active infection were administered in 24 patients (11.0%) with comparable rates between groups (16.3% vs. 9.5%, *p* = 0.18).

PAO-positive patients demonstrated significantly more complex intraoperative courses. Operative time was prolonged (median 52 vs. 42 min, *p* < 0.001), with 36.7% requiring >60 min versus 14.2% in the PAO-negative group (*p* < 0.001). Estimated blood loss was greater (35.2 ± 18.6 vs. 26.8 ± 13.9 mL, *p* = 0.004), with 24.5% exceeding 50 mL versus 9.5% (*p* = 0.007). The extent of internal sphincter incision was more extensive, both in absolute length (3.4 ± 1.1 vs. 2.6 ± 0.8 cm, *p* < 0.001) and percentage of circumference (42.6 ± 14.2% vs. 33.1 ± 11.3%, *p* < 0.001); incision >50% circumference was required in 36.7% versus 11.8% (*p* < 0.001).

Seton management was correspondingly more complex in PAO-positive patients: tightening setons were used more frequently (28.6% vs. 10.7%, *p* = 0.003), multiple setons were required in 20.4% versus 8.3% (*p* = 0.018), and time to removal was prolonged (median 35 vs. 26 days, *p* = 0.008), with 44.9% requiring >35 days versus 17.8% (*p* < 0.001). Tightening setons were preferentially selected for anatomically complex cases, as evidenced by higher rates of horseshoe extension, multiple tracts, and supralevator involvement in this group (Supplementary Table 1). Postoperative antibiotic duration was longer (median 7 vs. 5 days, *p* = 0.012), reflecting infectious complications requiring extended therapy. Seton material and intraoperative complication rates did not differ between groups (Table 2).

**Table 2:** Perioperative surgical characteristics.

Variable	Total (*n* = 218)	PAO-positive (*n* = 49)	PAO-negative (*n* = 169)	*p*-value
Operative time, minutes, median (IQR)	45 (35–58)	52 (42–68)	42 (34–52)	<0.001
Operative time >60 min, *n* (%)	42 (19.3)	18 (36.7)	24 (14.2)	<0.001
Estimated blood loss, mL, mean ± SD	28.5 ± 15.2	35.2 ± 18.6	26.8 ± 13.9	0.004
Blood loss >50 mL, *n* (%)	28 (12.8)	12 (24.5)	16 (9.5)	0.007
Internal sphincter incision				
Length, cm, mean ± SD	2.8 ± 0.9	3.4 ± 1.1	2.6 ± 0.8	<0.001
Circumference, %, mean ± SD	35.2 ± 12.6	42.6 ± 14.2	33.1 ± 11.3	<0.001
>50% circumference, *n* (%)*	38 (17.4)	18 (36.7)	20 (11.8)	<0.001
Seton placement				
Seton type, *n* (%)				0.003
Loose seton	186 (85.3)	35 (71.4)	151 (89.3)	
Tightening seton	32 (14.7)	14 (28.6)	18 (10.7)	
Seton material, *n* (%)				0.34
Silastic vessel loop	158 (72.5)	38 (77.6)	120 (71.0)	
Silk suture	60 (27.5)	11 (22.4)	49 (29.0)	
Multiple setons placed, *n* (%)	24 (11.0)	10 (20.4)	14 (8.3)	0.018
Postoperative course				
Postoperative antibiotics, days, median (IQR)	5 (4–7)	7 (5–9)	5 (4–6)	0.012
Hospital stay, days, median (IQR)	4 (3–6)	5 (4–7)	4 (3–5)	0.08
Time to seton removal, days, median (IQR)	28 (21–35)	35 (28–42)	26 (20–32)	0.008
Time to seton removal >35 days, *n* (%)	52 (23.9)	22 (44.9)	30 (17.8)	<0.001
Intraoperative complications, *n* (%)	8 (3.7)	4 (8.2)	4 (2.4)	0.068
Preoperative antibiotics for active infection, *n* (%)	24 (11.0)	8 (16.3)	16 (9.5)	0.18
Preoperative antibiotic duration, days, median (IQR)	5 (3–7)	7 (5–10)	5 (3–7)	0.12
Postoperative IV antibiotics, days, median (IQR)	2 (2–2)	2 (2–3)	2 (2–2)	0.08
Postoperative oral antibiotics, days, median (IQR)	5 (4–7)	7 (5–9)	5 (4–6)	0.012
Postoperative antibiotics, days, median (IQR)	5 (4–7)	7 (5–9)	5 (4–6)	0.012

PAO, postoperative adverse outcome; IQR, interquartile range. *Incision >50% circumference was performed in complex cases requiring extended drainage (horseshoe extension, supralevator involvement, or dense fibrosis) despite the standard limit of ≤40%.

### 3.3 Postoperative adverse outcomes

Within 6 months postoperatively, 49 patients (22.5%) experienced at least one adverse outcome: recurrence in 16 (7.3%), moderate-to-severe incontinence (Wexner ≥7) in 15 (6.9%), delayed wound healing (>12 weeks) in 22 (10.1%), and major complications (Clavien-Dindo ≥III) in 8 (3.7%). Five patients (2.3%) had two concurrent adverse outcomes, and two (0.9%) had three. Recurrence presented at a median of 4.2 months (IQR 2.8–5.6); 12 were MRI-confirmed and 6 diagnosed surgically. Among incontinent patients, 8 (53.3%) had grade I–II internal sphincter defects on endoanal ultrasound. Major complications comprised perianal abscess requiring drainage (*n* = 4), severe bleeding requiring transfusion (*n* = 2), and fecal impaction requiring hospitalization (*n* = 2).

Protocol compliance was assessable in 203 patients (93.1%) who completed diaries: 87.2% completed ≥90% of prescribed sitz baths, 82.8% maintained fiber supplementation for 4 weeks, and 71.4% achieved complete adherence to all six protocol components (Table 3).

**Table 3:** Incidence of postoperative adverse outcomes within 6 months.

Outcome	*n*	% (95% CI) or %*
Primary composite outcome (PAO)		
No adverse outcome	169	77.5 (71.6–82.7)
Any adverse outcome	49	22.5 (17.3–28.4)
Individual components (percentages based on total cohort, *n* = 218)		
Recurrence	16	7.3 (4.2–11.6)
Anal incontinence (Wexner score ≥7)	15	6.9 (3.9–11.1)
Delayed wound healing (>12 weeks)	20	9.2 (5.7–13.8)
Major complications (Clavien-Dindo ≥III)	7	3.2 (1.3–6.5)
Subclassification of outcomes (percentages based on those with the specific outcome)		
Among those with recurrence (*n* = 16):		
Confirmed by MRI	11	68.8*
Clinical diagnosis	5	31.3*
Time to recurrence, months, median (IQR)		4.2 (2.8–5.6)
Among those with incontinence (*n* = 15):		
With sphincter defect on EAUS	8	53.3*
Among those with major complications (*n* = 7):		
Perianal abscess requiring drainage	4	57.1*
Severe bleeding requiring transfusion	2	28.6*
Fecal impaction requiring hospitalization	1	14.3*
Multiple adverse outcomes		
Two concurrent outcomes	5	2.3 (0.7–5.2)
Three concurrent outcomes	2	0.9 (0.1–3.2)

PAO, postoperative adverse outcome; MRI, magnetic resonance imaging; EAUS, endoanal ultrasound; CI, confidence interval. *Percentages calculated among those with the specific parent outcome (e.g., 68.8% = 11/16 among those with recurrence); these are descriptive proportions and do not represent incidence in the total cohort, hence 95% CIs are not provided.

### 3.4 Risk factors for postoperative adverse outcomes

**Figure 2:**
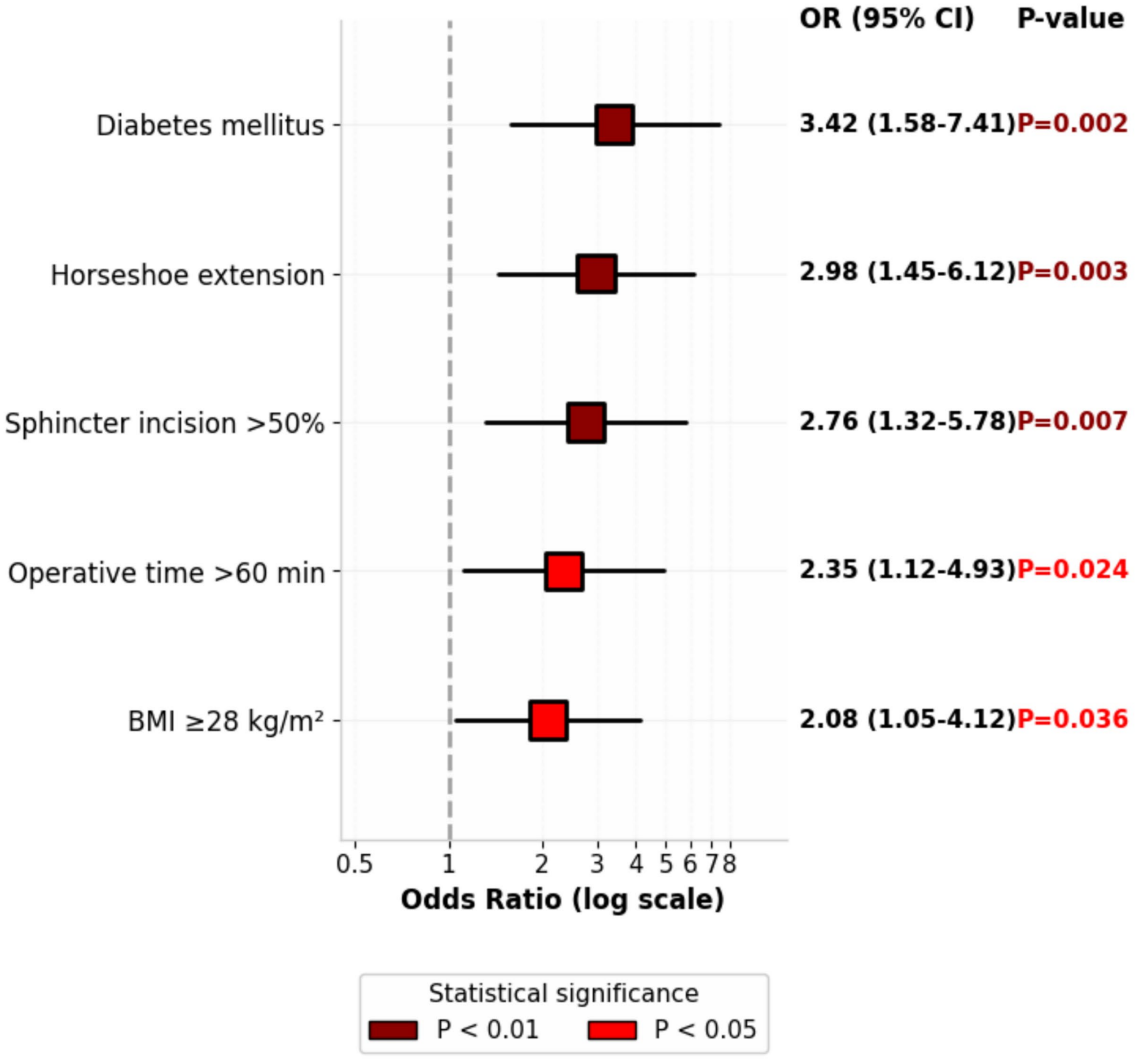
Forest plot of independent risk factors.

In the logistic regression model, PAO was treated as a binary variable (presence vs. absence of any adverse outcome), with patients experiencing multiple concurrent events counted as a single positive outcome. Multivariate logistic regression analysis identified five independent risk factors for postoperative adverse outcomes (Supplementary Table 2). Diabetes mellitus was the strongest predictor (adjusted OR 3.42, 95% CI 1.58–7.41, *p* = 0.002), followed by horseshoe fistula extension (adjusted OR 2.98, 95% CI 1.45–6.12, *p* = 0.003), internal sphincter incision >50% circumference (adjusted OR 2.76, 95% CI 1.32–5.78, *p* = 0.007), operative time >60 min (adjusted OR 2.35, 95% CI 1.12–4.93, *p* = 0.024), and BMI ≥ 28 kg/m^2^ (adjusted OR 2.08, 95% CI 1.05–4.12, *p* = 0.036, Figure 2). Parks classification (transsphincteric vs. suprasphincteric) was not selected in the final model because it showed no significant association with PAO in univariate analysis (*p* = 0.28, Table 1) and was collinear with Garg grade and internal opening height, which together captured anatomical complexity more granularly. Given the limited EPV ratio (7.0), wide confidence intervals were observed for some associations, particularly BMI ≥ 28 kg/m^2^ (OR 2.08, 95% CI 1.05–4.12), reflecting statistical uncertainty in these estimates. These adjusted ORs should be interpreted as hypothesis-generating rather than definitive effect estimates.

The final multivariate model demonstrated acceptable discrimination with a C-statistic of 0.82 (95% CI 0.76–0.88) and adequate calibration (Hosmer-Lemeshow test, *p* = 0.42). No significant multicollinearity was detected (all VIF values <5). Interaction analysis revealed no significant effect modification between diabetes and operative time (*p* = 0.18) or between horseshoe extension and sphincter incision extent (*p* = 0.31). When seton type was added to the multivariable model, it was not an independent predictor (adjusted OR 1.42, 95% CI 0.68–2.96, *p* = 0.35) after adjustment for horseshoe extension and sphincter incision extent, confirming that its apparent association with PAO was driven by indication bias. The association between operative time >60 min and PAO did not differ significantly between the two surgeons (*p* for interaction = 0.91), suggesting operative time reflects case complexity rather than surgeon-specific factors. Seton type was not independently predictive (OR 1.42, *p* = 0.35) after adjustment for complexity factors, suggesting confounding by indication.

### 3.5 Prediction model performance

**Figure 3:**
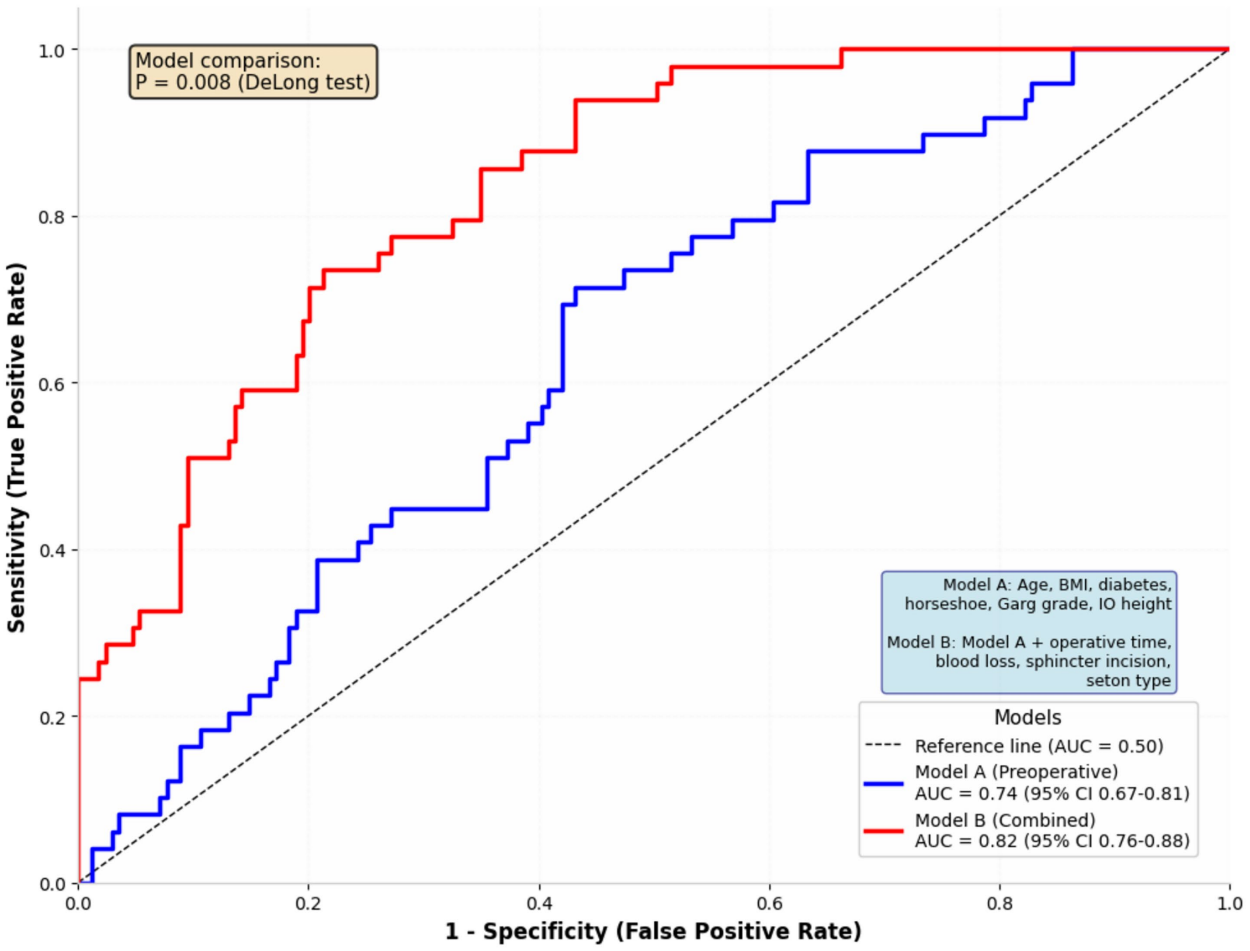
ROC curves for prediction models.

The preoperative model (Model A: age, BMI, diabetes, horseshoe extension, Garg grade, internal opening height) achieved a C-statistic of 0.74 (95% CI 0.67–0.81). The combined model (Model B: adding operative time, blood loss, sphincter incision extent, and seton type) significantly improved discrimination to 0.82 (95% CI 0.76–0.88; *p* = 0.008 for comparison with Model A, Figure 3). Bootstrap validation (B = 1,000) yielded an optimism-corrected C-statistic of 0.80 for Model B. Decision curve analysis demonstrated that using Model B to guide clinical decision-making would result in a net benefit of 0.18 at a threshold probability of 20%, corresponding to avoiding 18 adverse outcomes per 100 patients without increasing unnecessary interventions (Supplementary Figure 1).

Risk stratification using Model B identified 68 patients (31.2%) as high-risk (predicted probability >30%), with an observed adverse outcome rate of 41.2% (28/68), compared to 14.0% (21/150) in the low-risk group (predicted probability ≤30%; *p* < 0.001). A coefficient-weighted scoring system based on regression coefficients demonstrated equivalent discrimination to this simple count model (C-statistic 0.82 vs. 0.81) with identical risk classification for 94% of patients (Supplementary Table 3).

### 3.6 Functional outcomes

Baseline Wexner incontinence scores were comparable between groups (median 3 [IQR 2–4] vs. 3 [IQR 2–4], *p* = 0.72). At 6 months, the PAO-positive group demonstrated significantly worse continence (median 7 [IQR 5–9] vs. 3 [IQR 2–4], *p* < 0.001), with 30.6% developing moderate-to-severe incontinence versus none in the PAO-negative group. Anorectal manometry confirmed corresponding functional deterioration: anal resting pressure decreased from 58.2 ± 14.6 to 42.8 ± 12.3 mmHg (*p* < 0.001) and maximal squeeze pressure from 128.4 ± 32.5 to 98.6 ± 28.4 mmHg (*p* < 0.001). By contrast, the PAO-negative group showed preserved resting pressure (58.6 ± 15.2 vs. 56.4 ± 14.8 mmHg, *p* = 0.18) and modest squeeze pressure reduction (130.2 ± 34.1 vs. 118.6 ± 31.2 mmHg, *p* = 0.002).

To validate self-reported outcomes, we examined correlations between changes in Wexner scores and manometric parameters. In the total cohort, Wexner score change demonstrated moderate negative correlation with resting pressure change (r = −0.62, 95% CI − 0.71 to −0.51, *p* < 0.001) and squeeze pressure change (r = −0.58, 95% CI − 0.68 to −0.46, *p* < 0.001). While this supports the convergent validity of patient-reported continence assessment, the moderate rather than strong correlation underscores the inherent limitations of subjective outcome measures and the non-blinded assessment design.

Quality of life was significantly impaired in the PAO-positive group (QLAF-QS median 68 [IQR 52–78] vs. 88 [IQR 78–94], *p* < 0.001). Wound healing was prolonged (median 56 days [IQR 42–78] vs. 28 days [IQR 21–35], *p* < 0.001), and patient satisfaction was lower (median 6 [IQR 4–7] vs. 8 [IQR 7–9], *p* < 0.001) (Table 4).

**Table 4:** Functional outcomes and quality of life at 6 months postoperatively.

Outcome	PAO-positive (*n* = 49)	PAO-negative (*n* = 169)	*p*-value
Wexner incontinence score			
Preoperative, median (IQR)	3 (2–4)	3 (2–4)	0.72
6-month postoperative, median (IQR)	7 (5–9)	3 (2–4)	<0.001
Change from baseline, median (IQR)	+4 (+2 to +6)	0 (−1 to +1)	<0.001
Patients with moderate–severe incontinence (≥7), *n* (%)	15 (30.6)	0 (0)	<0.001
Anorectal manometry			
Anal resting pressure, mmHg			
Preoperative, mean ± SD	58.2 ± 14.6	58.6 ± 15.2	0.88
6-month postoperative, mean ± SD	42.8 ± 12.3	56.4 ± 14.8	<0.001
Change from baseline, mean ± SD	−15.4 ± 8.2	−2.2 ± 6.8	<0.001
Maximal squeeze pressure, mmHg			
Preoperative, mean ± SD	128.4 ± 32.5	130.2 ± 34.1	0.76
6-month postoperative, mean ± SD	98.6 ± 28.4	118.6 ± 31.2	<0.001
Change from baseline, mean ± SD	−29.8 ± 15.6	−11.6 ± 12.4	<0.001
Sphincter functional length, cm			
Preoperative, mean ± SD	3.8 ± 0.6	3.9 ± 0.7	0.42
6-month postoperative, mean ± SD	3.2 ± 0.8	3.7 ± 0.6	0.008
Quality of life (QLAF-QS)			
6-month score, median (IQR)	68 (52–78)	88 (78–94)	<0.001
Other outcomes			
Wound healing time, days, median (IQR)	56 (42–78)	28 (21–35)	<0.001
Patient satisfaction score (1–10), median (IQR)	6 (4–7)	8 (7–9)	<0.001
Return to normal activity, days, median (IQR)	21 (14–28)	14 (10–18)	<0.001

PAO, postoperative adverse outcome; QLAF-QS, Quality of Life Assessment for Fistula Questionnaire Score; IQR, interquartile range. Within-group comparisons performed using paired *t*-test or Wilcoxon signed-rank test. Between-group comparisons performed using independent *t*-test or Mann–Whitney U test.

### 3.7 Subgroup analysis

Stratified analysis by Garg grade revealed that the effect of diabetes on adverse outcomes was most pronounced in grade IIIA fistulas (adjusted OR 4.52, 95% CI 1.68–12.18, *p* = 0.003) compared to grade IIIB (adjusted OR 2.84, 95% CI 1.12–7.21, *p* = 0.028) and grade IV (adjusted OR 2.15, 95% CI 0.58–7.98, *p* = 0.25). The impact of horseshoe extension was consistent across all grades (*P* for interaction = 0.42).

**Figure 4:**
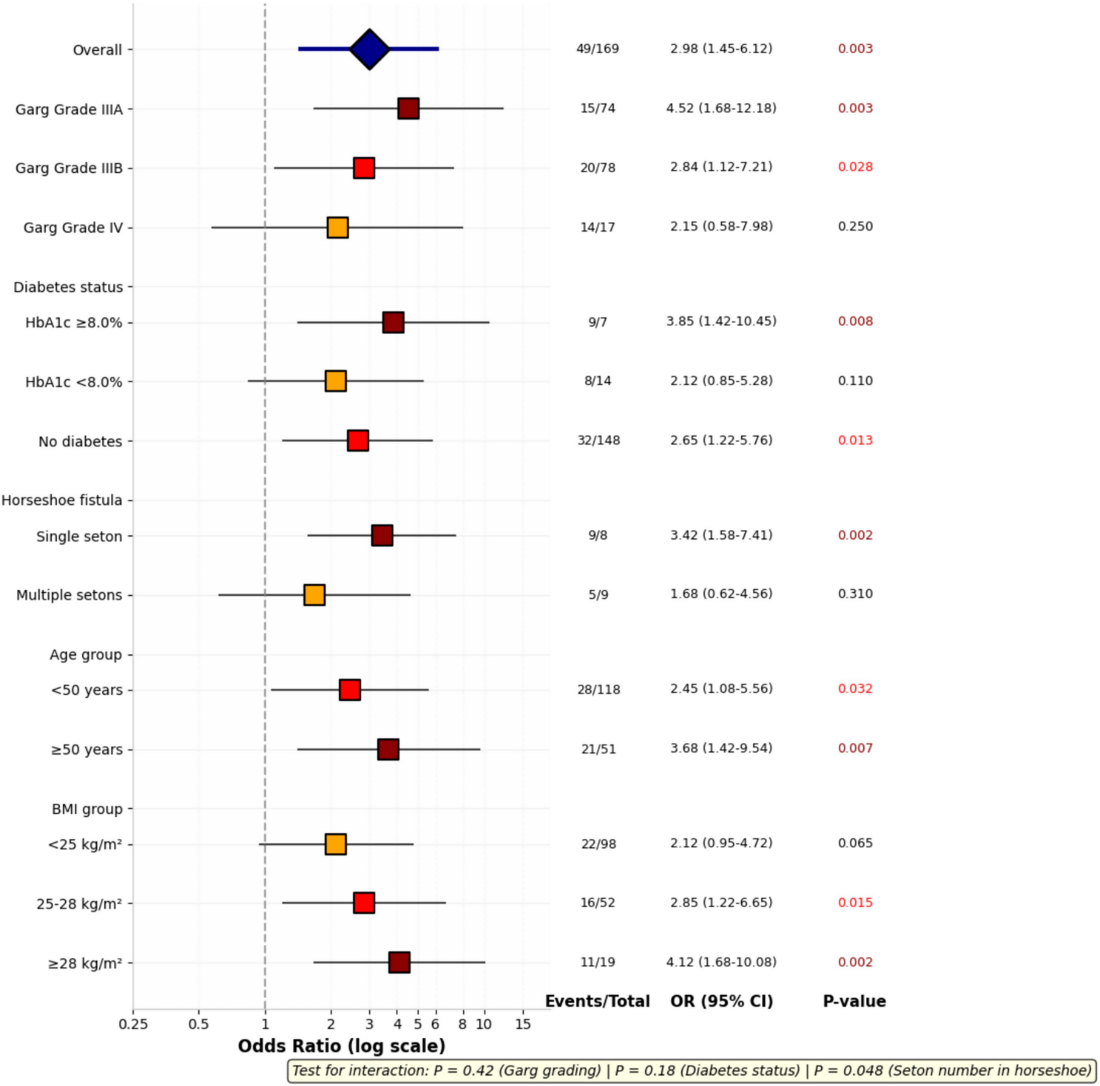
The association within different subgroups.

Among patients with diabetes (*n* = 38), a dose–response was observed: HbA1c ≥ 8.0 56.3%, HbA1c < 8.0 22.7%, non-diabetic 12.4% (*p* for trend <0.001). In patients with horseshoe fistulas (*n* = 47), combined use of multiple setons was associated with reduced adverse outcomes compared to single seton placement (26.3% vs. 52.9%, *p* = 0.048, Figure 4).

### 3.8 Sensitivity analyses

Using an alternative definition of incontinence (Wexner score ≥5), the multivariate model remained robust with similar effect sizes for all independent risk factors. Excluding diabetic patients (*n* = 180) attenuated the association between BMI ≥ 28 kg/m^2^ and adverse outcomes (adjusted OR 1.68, 95% CI 0.82–3.44, *p* = 0.16), while horseshoe extension (adjusted OR 2.84, 95% CI 1.32–6.12, *p* = 0.008) and sphincter incision >50% (adjusted OR 2.45, 95% CI 1.12–5.36, *p* = 0.025) remained significant predictors. Complete case analysis (*n* = 203) yielded results consistent with the primary analysis.

When incontinence was excluded from the composite endpoint (PAO-minus-incontinence: recurrence, delayed healing, or major complications), 34 patients (15.6%) experienced adverse outcomes. The risk factor profile remained consistent with the primary analysis: diabetes mellitus (adjusted OR 3.15, 95% CI 1.38–7.21, *p* = 0.006), horseshoe extension (OR 2.84, 95% CI 1.32–6.12, *p* = 0.008), internal sphincter incision >50% (OR 2.52, 95% CI 1.15–5.51, *p* = 0.021), and operative time >60 min (OR 2.18, 95% CI 1.02–4.67, *p* = 0.044) remained significant independent predictors. BMI ≥ 28 kg/m^2^ showed attenuated significance (OR 1.89, 95% CI 0.91–3.92, *p* = 0.089). Model discrimination was preserved (C-statistic 0.80, optimism-corrected 0.78) (Supplementary Table 4).

## 4 Discussion

In this retrospective cohort study of 218 patients undergoing modified TROPIS combined with Parks’ fistulotomy with seton for high anal fistula, 22.5% experienced adverse postoperative outcomes within 6 months, including recurrence (8.3%), incontinence (6.9%), delayed healing (10.1%), and major complications (3.7%). Multivariable analysis identified five independent predictors: diabetes mellitus (strongest, OR 3.42), horseshoe extension, extensive internal sphincter incision (>50%), prolonged operative time (>60 min), and obesity (BMI ≥ 28 kg/m^2^). The preoperative-intraoperative combined model demonstrated good discrimination (C-statistic 0.82) and clinical utility for risk stratification. These findings underscore modifiable and non-modifiable determinants of treatment failure in this sphincter-preserving approach, informing surgical planning and shared decision-making.

The predominant influence of diabetes mellitus on adverse outcomes likely reflects its multifaceted impact on wound healing. Hyperglycemia impairs neutrophil chemotaxis and bacterial clearance (11), predisposing to persistent infection and abscess recurrence, evident in our 34.7% diabetes prevalence among adverse outcome cases versus 12.4% without. Additionally, advanced glycation end-product accumulation cross-links collagen and impairs extracellular matrix remodeling (12, 13), directly compromising fistula tract epithelialization and explaining the 10.1% delayed healing rate observed. Critically, our subgroup analysis reveals that glycemic control substantially modifies this risk: patients with HbA1c ≥ 8.0% experienced 56.3% adverse outcome rates versus 22.7% in those with better control (*p* = 0.031). This dose–response relationship supports causality and identifies a modifiable therapeutic target. Preoperative optimization to HbA1c < 7.0% for 8–12 weeks—aligning with American Diabetes Association perioperative guidelines—may reduce infection risk by 30–40% based on observational data from abdominal surgery (14, 15). However, the optimal glycemic target and optimization duration specifically for perineal surgery remain undefined, representing an urgent research priority given the high prevalence of diabetes in our cohort (17.4%) and its disproportionate impact on outcomes.

Horseshoe extension emerged as the second strongest predictor (OR 2.98), reflecting pathophysiological mechanisms distinct from simple fistulas. The circumferential involvement of the anal canal creates a chronic inflammatory microenvironment characterized by: (i) bacterial persistence and biofilm formation, with posterior tracts often harboring secondary extensions that evade immune clearance despite preoperative MRI (sensitivity 70–85% for horseshoe complexity) (16), leading to sustained infection and abscess recurrence; (ii) compromised tissue oxygenation and perfusion, as extensive inflammatory involvement and surgical dissection disrupt the rich anastomotic network of the posterior anal canal, impairing collagen synthesis and epithelialization (17, 18); and (iii) cumulative sphincter injury, where the circumferential course necessitates more extensive division of muscle fibers than hemispheric fistulas, predisposing to internal sphincter dysfunction and fecal incontinence (19, 20).

Operative time >60 min should be interpreted as a surrogate marker of anatomical complexity rather than a modifiable technical variable. Prolonged surgery reflects unmeasured intraoperative challenges—extensive fibrosis, multiple tracts, or difficult sphincter dissection (21)—that are not fully captured by preoperative imaging. This interpretation is supported by three observations in our data: (i) operative time was not predictive in simple Garg grade IIIA fistulas (subgroup analysis); (ii) no surgeon-specific effect was detected (*p* for interaction = 0.91); and (iii) blood loss >50 mL, another complexity proxy, showed collinearity with operative time (VIF = 3.2) and was not independently selected. Clinically, operative time should not be used as a quality metric; instead, prolonged cases should trigger postoperative enhanced surveillance. This is supported by the absence of operative time as a predictor in simpler fistulas (Garg grade IIIA) in our subgroup analysis, suggesting that operative time thresholds should be anatomy-stratified: 60 min may be an appropriate cutoff for complex horseshoe fistulas, whereas in straightforward cases, this threshold may be overly conservative (22). Beyond these technical considerations, the seton has demonstrated renewed clinical utility during healthcare disruptions such as the COVID-19 pandemic, serving as a temporizing measure when definitive surgery was delayed (23).

The association between extensive internal sphincter incision (>50% circumference) and adverse outcomes (OR 2.76) encapsulates the fundamental tension in complex fistula surgery. Our finding validates the “less is more” philosophy underlying sphincter-preserving techniques (24), but also reveals practical constraints: 17.4% of patients required >50% incision due to anatomical necessity (horseshoe extension, supralevator involvement, dense fibrosis). This creates a therapeutic paradox: inadequate division risks recurrence from insufficient drainage, while aggressive division compromises continence (25, 26). The independent predictive value of >50% incision—despite being reserved for the most complex cases—suggests that anatomical indications for extension may themselves exert residual risk, or that functional consequences of extensive division are not fully mitigated by the sphincter-preserving intent (19). Preoperative decision algorithms incorporating MRI-predicted incision extent (e.g., advancement flap when >40% anticipated) may resolve this tension, though prospective validation is required (27).

Our findings contextualize within evolving benchmarks for complex fistula surgery. The 22.5% composite adverse outcome rate exceeds reported rates for TROPIS alone (15–20%) and LIFT (12–18%) (5, 7), reflecting three design features: (i) broader outcome definition incorporating incontinence and delayed healing, not merely recurrence; (ii) longer follow-up (6 vs. 3 months) capturing delayed failures; and (iii) transsphincteric complexity managed with seton rather than simple LIFT. Notably, our 8.3% recurrence rate compares favorably with traditional fistulotomy (15–30%) (4), supporting the sphincter-preserving rationale, while the 6.9% incontinence rate—though lower than historic fistulotomy—remains clinically significant in our young cohort (mean 42.6 years) with decades of follow-up ahead.

Methodologically, our emphasis on composite outcomes and risk prediction models addresses gaps in prior literature. Most studies examine recurrence or incontinence in isolation (28), potentially overlooking interconnected failure modes: our data show 14.3% of adverse outcomes involved multiple domains (e.g., recurrence with delayed healing), suggesting shared pathophysiology (poor wound healing to persistent tract to recurrence). The prediction model (C-statistic 0.82, optimism-corrected 0.80) offers clinical utility superior to single-factor decision-making, with decision curve analysis demonstrating net benefit at 20% risk threshold. Given the equivalent discrimination of coefficient-weighted and equal-count risk stratification, we retained the simpler equal-weight approach for bedside practicality.

These findings enable practical risk stratification: 0–1 factors (~45%, risk <15%): standard care; 2–3 factors (~40%, risk 25–35%): optimize HbA1c, prefer loose seton; ≥4 factors (~15%, risk >50%): consider flap/LIFT or referral. External validation is needed. Prospective validation of this algorithm, particularly the HbA1c threshold and high-risk procedure selection, is needed before quality metric implementation.

We excluded 8 patients (3.1%) with previous anal surgery for the same fistula to ensure homogeneous anatomy and avoid confounding from surgical scarring. This improves internal validity but limits generalizability: in clinical practice, 30–50% of complex fistulas present as recurrences after failed prior procedures (29). Recurrent fistulas typically exhibit more complex anatomy and 1.5- to 2-fold higher adverse outcome rates due to compromised tissue vascularity, distorted surgical planes, and impaired wound healing (28). Our findings therefore likely represent a “best-case” estimate for primary fistulas; application to recurrent cases should be tempered accordingly. Prospective studies specifically examining modified TROPIS-plus-seton outcomes in this challenging subgroup are warranted.

Despite these methodological strengths, several limitations temper our conclusions. The EPV ratio of 7.0 falls below the conventional threshold for stable logistic regression, and while Firth’s penalized likelihood mitigates bias, effect estimate precision remains constrained (e.g., BMI 95% CI 1.05–4.12). We therefore emphasize risk factor identification—where diabetes and horseshoe extension emerge as robust predictors—over definitive individual risk prediction, pending external validation. Residual confounding likely affects operative time (correlating with unmeasured complexity such as fibrosis density and operator experience) and seton type (tightening setons were selected for more complex cases, potentially masking protective effects of loose setons). Additionally, functional outcomes were assessed by assessor-blinded rather than double-blind design, as patients inherently knew their symptoms. The moderate correlation (r = −0.58 to −0.62) between Wexner score changes and manometric parameters, while supporting convergent validity, also highlights the limitations of patient-reported outcomes in the absence of true blinding. Generalizability is limited to high-volume tertiary centers with standardized protocols, and 6-month follow-up, while capturing early failures, may miss delayed incontinence or quality-of-life deterioration in this young cohort.

## 5 Conclusion

Modified TROPIS combined with Parks’ fistulotomy with seton for high anal fistula is associated with a 22.5% rate of adverse postoperative outcomes, driven by diabetes, horseshoe extension, extensive sphincter division, prolonged surgery, and obesity. An internally validated risk prediction model demonstrated promising discrimination and requires external validation before implementation for clinical risk stratification. These findings support the continued use of this combined technique while highlighting the critical importance of metabolic optimization, technical precision, and individualized care in complex fistula surgery. Future prospective studies should focus on model validation, comparative effectiveness, and strategies to mitigate modifiable risk factors.

## Data Availability

The original contributions presented in the study are included in the article/Supplementary material, further inquiries can be directed to the corresponding author.
