# Supplementary material for: Risk factors for postoperative adverse outcomes in patients with high anal fistula undergoing modified TROPIS procedure combined with Parks’ fistulotomy with seton: a retrospective study
Source: Front Med (Lausanne). 2026 Jun 1;13:1834676. doi: 10.3389/fmed.2026.1834676 (PMC13265489; doi:10.3389/fmed.2026.1834676)
Supplement: Supplementary file 1 [file Table_1.DOCX]

**Supplementary Table 1.** Baseline complexity comparison by seton type

| Variable | Tightening seton (n=32) | Loose seton (n=186) | P-value |
| --- | --- | --- | --- |
| Horseshoe extension, n (%) | 18 (56.3) | 29 (15.6) | <0.001 |
| Multiple tracts, n (%) | 16 (50.0) | 55 (29.6) | 0.018 |
| Supralevator extension, n (%) | 10 (31.3) | 18 (9.7) | 0.002 |
| Internal sphincter incision >50%, n (%) | 22 (68.8) | 16 (8.6) | <0.001 |
| Operative time >60 min, n (%) | 16 (50.0) | 26 (14.0) | <0.001 |
| PAO, n (%) | 14 (43.8) | 35 (18.8) | 0.003 |

## **Supplementary Table.2 Univariate and multivariate logistic regression analysis of risk factors for postoperative adverse outcomes**

| Variable | Univariate OR  (95% CI) | P-value | Multivariate OR  (95% CI) | P-value |
| --- | --- | --- | --- | --- |
| Age, years (≥50 vs <50) | 2.45 (1.28-4.68) | 0.007 | 1.68 (0.82-3.44) | 0.16 |
| BMI, kg/m² (≥28 vs <28) | 2.68 (1.42-5.06) | 0.002 | 2.08 (1.05-4.12) | 0.036 |
| Diabetes mellitus (yes vs no) | 3.78 (1.82-7.84) | <0.001 | 3.42 (1.58-7.41) | 0.002 |
| Smoking (current/former vs never) | 2.01 (1.02-3.96) | 0.044 | 1.45 (0.68-3.10) | 0.33 |
| Hypertension (yes vs no) | 2.02 (0.98-4.16) | 0.058 | — | — |
| Fistula duration, months (≥12 vs <12) | 2.12 (1.12-4.02) | 0.021 | 1.52 (0.74-3.12) | 0.25 |
| Previous perianal abscess (yes vs no) | 1.89 (1.02-3.50) | 0.043 | — | — |
| Parks' classification (supra vs trans) | 1.26 (0.64-2.48) | 0.51 | — | — |
| Garg grading (IV vs IIIA/IIIB) | 3.52 (1.58-7.84) | 0.002 | 1.88 (0.76-4.64) | 0.17 |
| Horseshoe extension (yes vs no) | 4.12 (2.08-8.16) | <0.001 | 2.98 (1.45-6.12) | 0.003 |
| Multiple fistula tracts (yes vs no) | 2.78 (1.48-5.22) | 0.001 | 1.65 (0.82-3.32) | 0.16 |
| Internal opening >3cm (yes vs no) | 2.65 (1.35-5.20) | 0.005 | 1.72 (0.84-3.52) | 0.14 |
| Supralevator extension (yes vs no) | 3.08 (1.38-6.88) | 0.006 | — | — |
| Operative time, minutes (>60 vs ≤60) | 3.25 (1.68-6.28) | <0.001 | 2.35 (1.12-4.93) | 0.024 |
| Blood loss, mL (>50 vs ≤50) | 2.45 (1.28-4.68) | 0.007 | — | — |
| Sphincter incision >50% (yes vs no) | 4.42 (2.18-8.96) | <0.001 | 2.76 (1.32-5.78) | 0.007 |
| Tightening seton (vs loose seton) | 3.35 (1.52-7.38) | 0.003 | 1.92 (0.82-4.50) | 0.13 |
| Multiple setons (yes vs no) | 2.82 (1.18-6.74) | 0.019 | — | — |
| Time to seton removal, days (>35 vs ≤35) | 2.55 (1.32-4.92) | 0.006 | — | — |

****Note:**** OR = odds ratio; CI = confidence interval; BMI = body mass index. Variables with P<0.10 in univariate analysis were entered into multivariate model. Final model: Hosmer-Lemeshow test P=0.42; C-statistic=0.82 (95% CI 0.76-0.88). — = variable not retained in final multivariate model.

**Supplementary Table 3**. Coefficient-weighted risk score

| **Risk factor** | **Adjusted OR** | **ln(OR)** | **Points** |
| --- | --- | --- | --- |
| Diabetes mellitus | 3.42 | 1.23 | 2 |
| Horseshoe extension | 2.98 | 1.09 | 1 |
| Internal sphincter incision >50% | 2.76 | 1.01 | 1 |
| Operative time >60 min | 2.35 | 0.85 | 1 |
| BMI ≥28 kg/m² | 2.08 | 0.73 | 1 |
| ****Total points**** | ****Risk category**** | ****n (%)**** | ****Observed PAO rate**** |
| 0–1 | Low | 98 (45.0%) | 13.3% |
| 2–3 | Intermediate | 87 (39.9%) | 28.7% |
| ≥4 | High | 33 (15.1%) | 51.5% |

Note: This weighted model demonstrated equivalent discrimination to the equal-count model (C-statistic 0.82 vs. 0.81) with identical risk classification for 94% of patients.

**Supplementary Table 4.** Sensitivity analysis: independent risk factors for composite endpoint excluding incontinence (recurrence, delayed healing, or major complications)

| Variable | Adjusted OR | 95% CI | P-value |
| --- | --- | --- | --- |
| Diabetes mellitus | 3.15 | 1.38–7.21 | 0.006 |
| Horseshoe extension | 2.84 | 1.32–6.12 | 0.008 |
| Internal sphincter incision >50% circumference | 2.52 | 1.15–5.51 | 0.021 |
| Operative time >60 minutes | 2.18 | 1.02–4.67 | 0.044 |
| BMI ≥28 kg/m² | 1.89 | 0.91–3.92 | 0.089 |

Model discrimination: C-statistic 0.80 (95% CI 0.73–0.87), optimism-corrected 0.78.


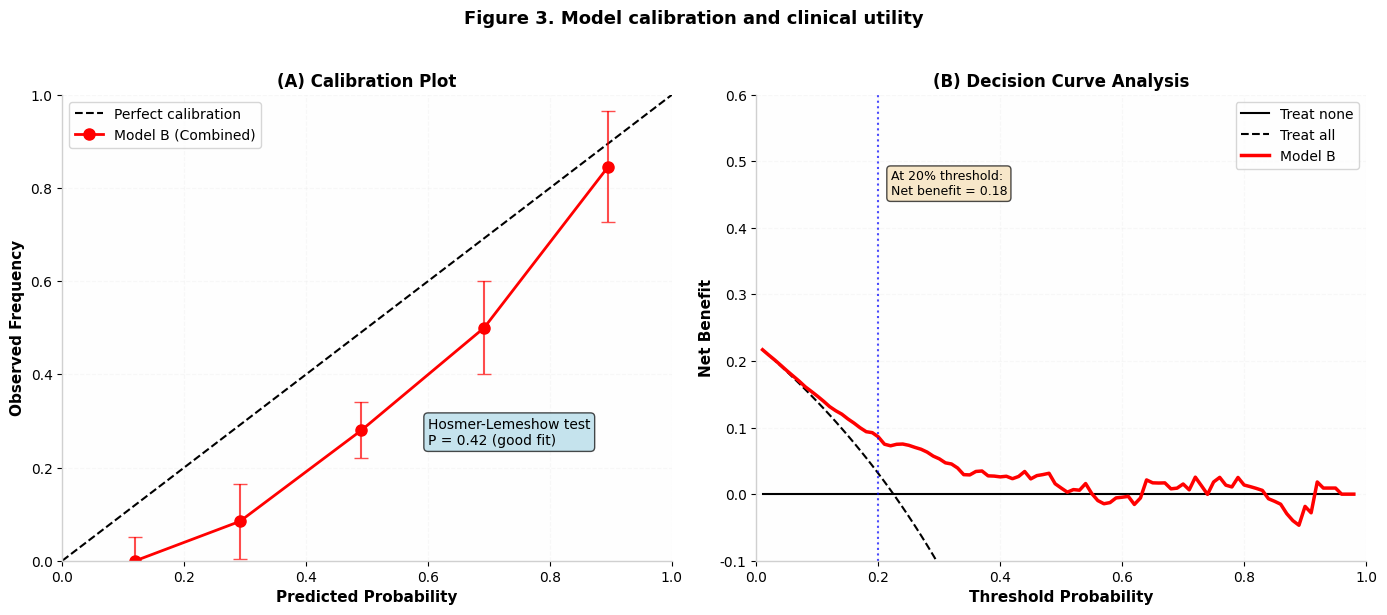


**Supplementary Figure.1** Model calibration and clinical utility
